# Long non-coding RNA RUNXOR accelerates MDSC-mediated immunosuppression in lung cancer

**DOI:** 10.1186/s12885-018-4564-6

**Published:** 2018-06-18

**Authors:** Xinyu Tian, Jie Ma, Ting Wang, Jie Tian, Yu Zheng, Rongrong Peng, Yungang Wang, Yue Zhang, Lingxiang Mao, Huaxi Xu, Shengjun Wang

**Affiliations:** 1 Department of Laboratory Medicine, The Affiliated People’s Hospital, Jiangsu University, Zhenjiang, 212012 China; 2 Institute of Laboratory Medicine, Jiangsu Key Laboratory of Laboratory Medicine, School of Medicine, Jiangsu University, Zhenjiang, China; 3 Department of Laboratory Medicine, Jiangsu Cancer Hospital, Nanjing, China

**Keywords:** lncRNA RUNXOR, MDSCs, RUNX1, Anti-tumor immunity, Lung cancer

## Abstract

**Background:**

RUNX1 overlapping RNA (RUNXOR) is a long non-coding RNA that has been indicated as a key regulator in the development of myeloid cells by targeting runt-related transcription factor 1 (RUNX1). Myeloid-derived suppressor cells (MDSCs) are a heterogeneous population of cells consisting of immature granulocytes and monocytes with immunosuppression. However, the impact of lncRNA RUNXOR on the development of MDSCs remains unknown.

**Methods:**

Both the expressions of RUNXOR and RUNX1 in the peripheral blood were measured by qRT-PCR. Human MDSCs used in this study were isolated from tumor tissue of patients with lung cancer by FCM or induced from PBMCs of healthy donors with IL-1β + GM-CSF. Specific siRNA was used to knockdown the expression of RUNXOR in MDSCs.

**Results:**

In this study, we found that the lncRNA RUNXOR was upregulated in the peripheral blood of lung cancer patients. In addition, as a target gene of RUNXOR, the expression of RUNX1 was downregulated in lung cancer patients. Finally, the expression of RUNXOR was higher in MDSCs isolated from the tumor tissues of lung cancer patients compared with cells from adjacent tissue. In addition, RUNXOR knockdown decreased Arg1 expression in MDSCs.

**Conclusions:**

Based on our findings, it is illustrated that RUNXOR is significantly associated with the immunosuppression induced by MDSCs in lung cancer patients and may be a target of anti-tumor therapy.

## Background

Lung cancer has become a leading cause of male cancer-related death worldwide because of its poor outcome and late diagnosis. Despite the development of chemotherapy and the integration of targeted therapy aimed at lung cancer, the overall outcomes are still not ideal [1]. Meanwhile, immunotherapy is becoming increasingly promising in the treatment of lung cancer [2, 3]. Nowadays, the immunosuppression induced by myeloid-derived suppressor cells (MDSCs) has been demonstrated to be a main cause of tumor escape in anti-tumor therapies targeting lung cancer [4–6].

MDSCs are a heterogeneous population of immature myeloid cells consisting of precursors for granulocytes, macrophages or dendritic cells (DCs), which accumulate during tumor progression [7, 8]. MDSCs display a broadly distinct phenotype. In mice, the phenotype of MDSCs is CD11b + Gr1+, which contain two subsets: polymorphonuclear MDSCs (PMN-MDSCs) characterized as CD11b + Ly6G + Ly6Clo and monocytic MDSCs (M-MDSCs) characterized as CD11b + Ly6G-Ly6Chi. In human, MDSCs represent a population of cells with the phenotype of CD11b + CD33 + HLA-DR-CD14-, which are further subdivided into PMN-MDSCs and M-MDSCs based on the differential expression of Lin and CD15 [9, 10]. In cancer progression, MDSCs inhibit the anti-tumor immune responses induced by CD4+ T cells, CD8+ T cells and NK cells by releasing Arg1, ROS and iNOS. In addition, MDSCs can also induce Treg cells and promote IL-10 production [11, 12]. Currently, therapies targeting MDSCs mainly involve eliminating these cells, inhibiting their suppressive effects or promoting their differentiation [13].

Functional genomics studies have revealed that ~ 90% of human genes produce non-coding RNAs (ncRNAs) consisting of long non-coding RNAs (> 200 nt) and microRNAs [14, 15]. Unlike microRNAs, lncRNAs are capable of being capped and polyadenylated. Increasing evidences indicate that lncRNAs are involved in different cellular processes via a variety of mechanisms [16–20]. The lncRNA RUNXOR, which is approximately 216 kb in length, is a long intragenic non-coding RNA. RUNXOR interacts epigenetically with the RUNX1 gene, which normally functions as a tumor suppressor and modulates the expression of a number of important hematopoietic regulator genes. LncRNA RUNXOR is unspliced and overlaps with RUNX1 introns and exons. In AML cells, RUNXOR regulates RUNX1 expression by directly binding to promoters and enhancers via its 3′-end and may be physically involved in chromosomal translocation that occur in malignancies. In addition, by directly binding to chromatin, RUNXOR is involved in the orchestration of a long-range intrachromosomal loop. The formation of intrachromosomal loop is a typical epigenetic mechanism by which a regulatory element can mediate the expression of a gene even when locate far away from the gene. The most remarkable property of RUNXOR is that this lncRNA is able to use its 3′-end to recruit RUNX1 protein to bind RUNX1 promoter and induce epigenetic modulation via a variety of enhancers [21]. In our previous study, we have demonstrated that miR-9 mediates the development of MDSCs by targeting RUNX1 [12]. Thus, in this study, we aimed to determine whether RUNXOR regulates the immunosuppression of MDSCs by targeting RUNX1 in the progression of lung cancer.

## Methods

### Patients and samples

One hundred peripheral blood samples which contained lung adenocarcinoma (*n* = 53), squamous cell lung cancer (*n* = 24) and small cell lung cancer (*n* = 23) were collected from lung cancer patients. To separate the cells from the plasma, we centrifuged peripheral blood samples at 20 °C and 2000 rpm for 5 min. Then ACK lysing buffer was used to lyse red blood cells, and the remaining cells were used in the subsequent experiments. Paired peripheral blood samples, pre- and post- surgery, were collected from 40 lung cancer patients. Paired lung cancer tissues and adjacent tissues were collected from 9 patients with lung cancer who underwent primary surgical resection. The study was approved by the respective Ethics Committee of the Affiliated People’s Hospital of Jiangsu University. Written informed consent was obtained from all the subjects in accordance with the Declaration of Helsinki.

### Isolation of MDSCs from tumor tissue

Collagenase II (Sigma-Aldrich, St. Louis, MO) was used to digest tumor tissue and adjacent tissue derived from patients with lung cancer that had been cut into small pieces (1–2 mm^3^) at 37 °C for 2 h on a rotating platform to obtain a single-cell suspension. The cells were collected and stained with human anti-HLA-DR, anti-CD33, anti-CD11b and anti-CD14 mAbs (eBioscience, San Diego, CA) for 30 min. Stained cells were collected and then analyzed via flow cytometry (FACSAria, BD Biosciences).

### Induction of human MDSCs

Density-gradient centrifugation over a Ficoll-Hypaque solution (Haoyang Biological Technology Co., Tianjin, China) was used to isolate human peripheral blood mononuclear cells (PBMCs). Then 40 ng/mL IL-1β (Peprotech, NJ) and 40 ng/mL GM-CSF (Peprotech) were used to stimulate PBMCs from healthy donors for 4 days. At day 4, the cells were collected and isolated by using human anti-CD33 beads (Miltenyi Biotec, Auburn, CA).

### Flow cytometry

To confirm the percentages of CD4+ and CD8+ T cells, 50 ng/ml phorbol myristate acetate (PMA; Sigma-Aldrich, California) and 1 μg/ml ionomycin (Sigma-Aldrich) were used to stimulate PBMCs from lung cancer patients and healthy donors for 2 h and then cells were incubated in the presence of 1 μg/ml brefeldin-A (eBioscience, San Diego) for another 4 h. Post stimulation, cells were then stained with human anti-CD3 and anti-CD8 mAbs (eBioscience), fixed, permeabilized and stained with a human anti-IFN-γ mAb (eBioscience) following the instructions of the Intracellular Staining Kit (Invitrogen, Carlsbad, CA).

### RNA isolation and quantitative real-time PCR

Total RNA was extracted from cells with TRIzol reagent (Invitrogen, California) following the manufacturer’s instructions. Random primers and a ReverTra Ace® qPCR RT Kit (Toyobo, Osaka, Japan) were used to synthesize cDNA. Bio-Rad SYBR Green Supermix (Bio-Rad, Hercules) was used to perform quantitative real-time PCR in triplicate. The primer sequences were as follows: human β-actin, sense 5-GAGTGTGGAGACCATCAAGGA-3, antisense 5-TGTATTGCTTTGCGTTGGAC-3; human RUNX1, sense 5-TGATGGCTGGCAATGATGAA-3, antisense 5-TGCGGTGGGTTTGTGAAGAC-3; human 18S, sense 5-CGGACAGGATTGACAGATTG-3, antisense 5- GCCAGAGTCTCGTTCGTTATC-3; human ARG1, sense 5-. CCTTTGCTGACATCCCTAAT-3, antisense 5-GATTCTTCCGTTCTTCTTGACT-3; and human RUNXOR, sense 5-CCTGTTCACGGTCCAAACTGG-3, antisense 5-CGGCAAGATCACAGTCCCTAGC-3. The expression level of each gene was expressed as the ratio to the β-actin transcript level. The data were analyzed with Bio-Rad CFX Manager software.

### Transfection

50 nM RUNXOR siRNA or its negative control (Ribobio Co., Guangzhou, China) was used to transfect MDSCs plated in 48-well plates according to the manufacturer’s protocol.

### Graphing and statistical analysis of data

To generate bar graphs or graphs of tumor regression, data from all experiments were entered into GraphPad Prism 5.0 (GraphPad, San Diego, CA). The data are presented as the mean ± SD. Student’s t-test was used to determine the statistical significance of differences between groups. And Spearman’s correlation coefficient was used to confirm correlations between variables. Differences were considered significant at a *p* level less than 0.05.

## Results

### The proportion of MDSCs is negatively correlated with the percentage of Th1/CTL cells in the peripheral blood of lung cancer patients

**Fig. 1:**
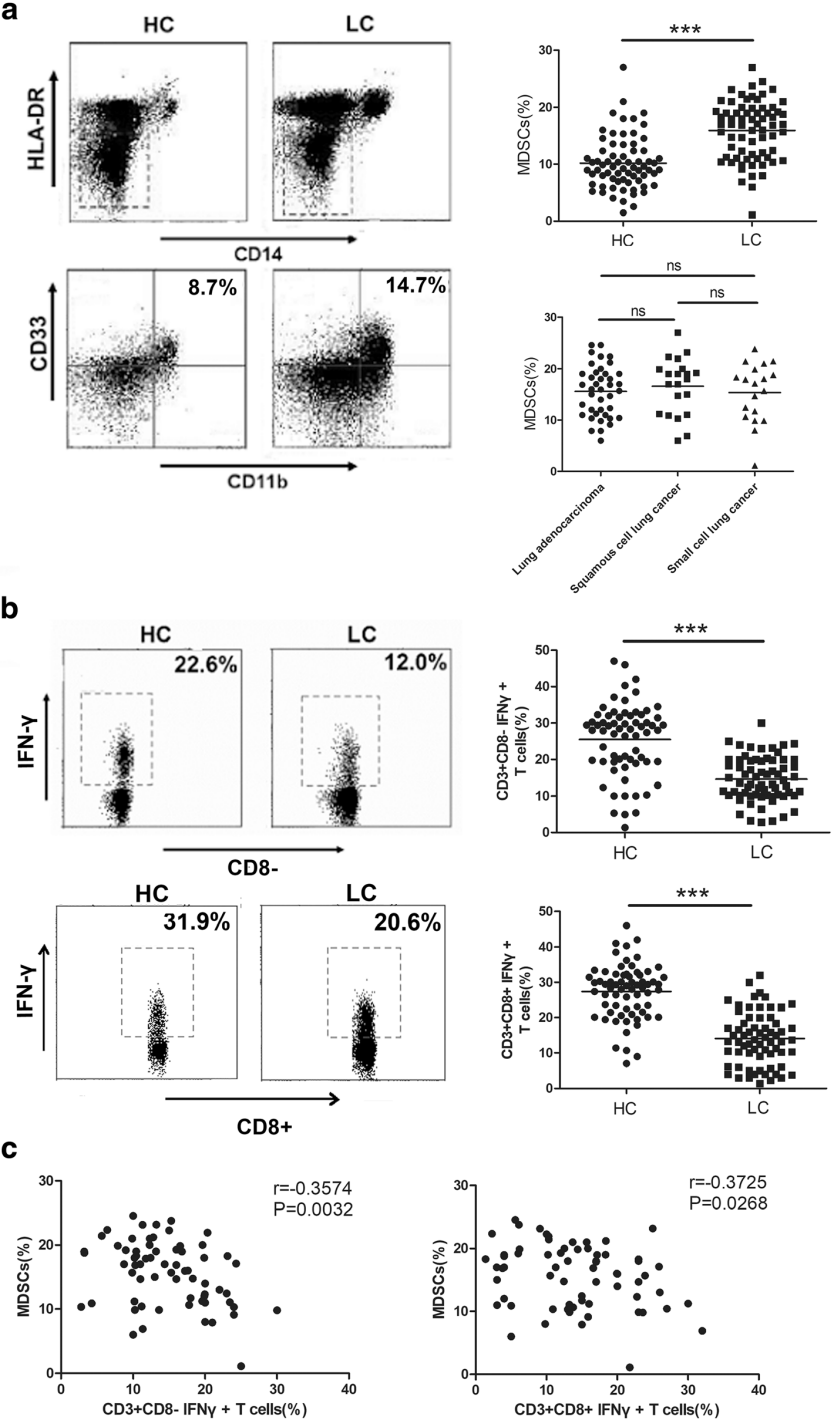
The percentage of MDSCs in the peripheral blood of lung cancer patients is negatively correlated with the ratio of Th1/CTL cells. **a**-**b** The proportions of CD11b + CD33 + HLA-DR-CD14- MDSCs (*n* = 61), CD3 + CD8-IFN-γ + Th1 cells and CD3 + CD8 + IFN-γ + CTLs (*n* = 56) in the peripheral blood of lung cancer patients and healthy donors were detected by flow cytometry (FCM). **c** The correlation between the proportion of Th1/CTL cells and the percentage of MDSCs in the peripheral blood of lung cancer patients. ***P < 0.001, **P < 0.01, ns = no significance

MDSCs are a population of cells that accumulate during the progression of various cancers. In human, the phenotype of MDSCs is CD11b + CD33 + HLA-DR-CD14-. These cells inhibit the anti-tumor immune response via different mechanisms: MDSCs produce suppressive molecules, such as Arg1, ROS or iNOS, to directly suppress the anti-tumor immune response induced by Th1/CTL cells and promote tumor progression; MDSCs can also promote the production of IL-10 to inhibit the CTL response by inducing Tregs or developing into tumor-associated macrophages (TAMs) [10, 22–25]. To determine whether the MDSCs proportion changes during lung cancer progression, we detected the percentage of MDSCs in the peripheral blood of healthy controls and lung cancer patients. Compared with the healthy controls, the proportion of MDSCs increased in the peripheral blood of lung cancer patients (*P* < 0.001, Fig. 1a). We also compared the proportion of MDSCs in the peripheral blood of lung cancer patients with different histological categories. The results indicated that the proportion of MDSCs in various histological categories showed no significant difference (Fig. 1a). At the same time, the proportions of Th1 cells and CTL cells decreased in lung cancer patients (*P* < 0.01, Fig. 1b). In the subsequent correlation analysis, we found that the proportion of MDSCs was significant negatively correlated with the percentage of Th1 or CTL (Fig. 1c) cells in the peripheral blood of lung cancer patients. These data suggest that MDSCs are a population of cells that inhibit Th1/CTL cells and induce anti-tumor immunity.

**Table 1:** Correlation between RUNXOR expression and the clinicopathological parameters of lung cancer patients

Relative expression of RUNXOR				
Parameter	Number	Low	High	*P*-value
Age				0.0294
< 60	33	8	25	
≥ 60	67	19	48	
Gender				0.3677
Male	74	22	52	
Female	26	6	20	
Tumor size				0.8746
≤ 3 cm	24	16	8	
> 3 cm	76	9	67	
Smoking history				0.039
Smokers	66	11	55	
Never smokers	34	17	17	
Lymph node metastasis				0.0028
Positive	67	9	58	
Negative	33	15	18	
TNM stage				< 0.0001
I + II	12	7	5	
III + IV	88	14	74	
Histological tumor type				0.016
Squamous cell carcinoma	53	14	39	
Adenocarcinoma	24	6	18	
Small cell lung cancer	23	9	14	

### LncRNA RUNXOR level is upregulated in lung cancer

**Fig. 2:**
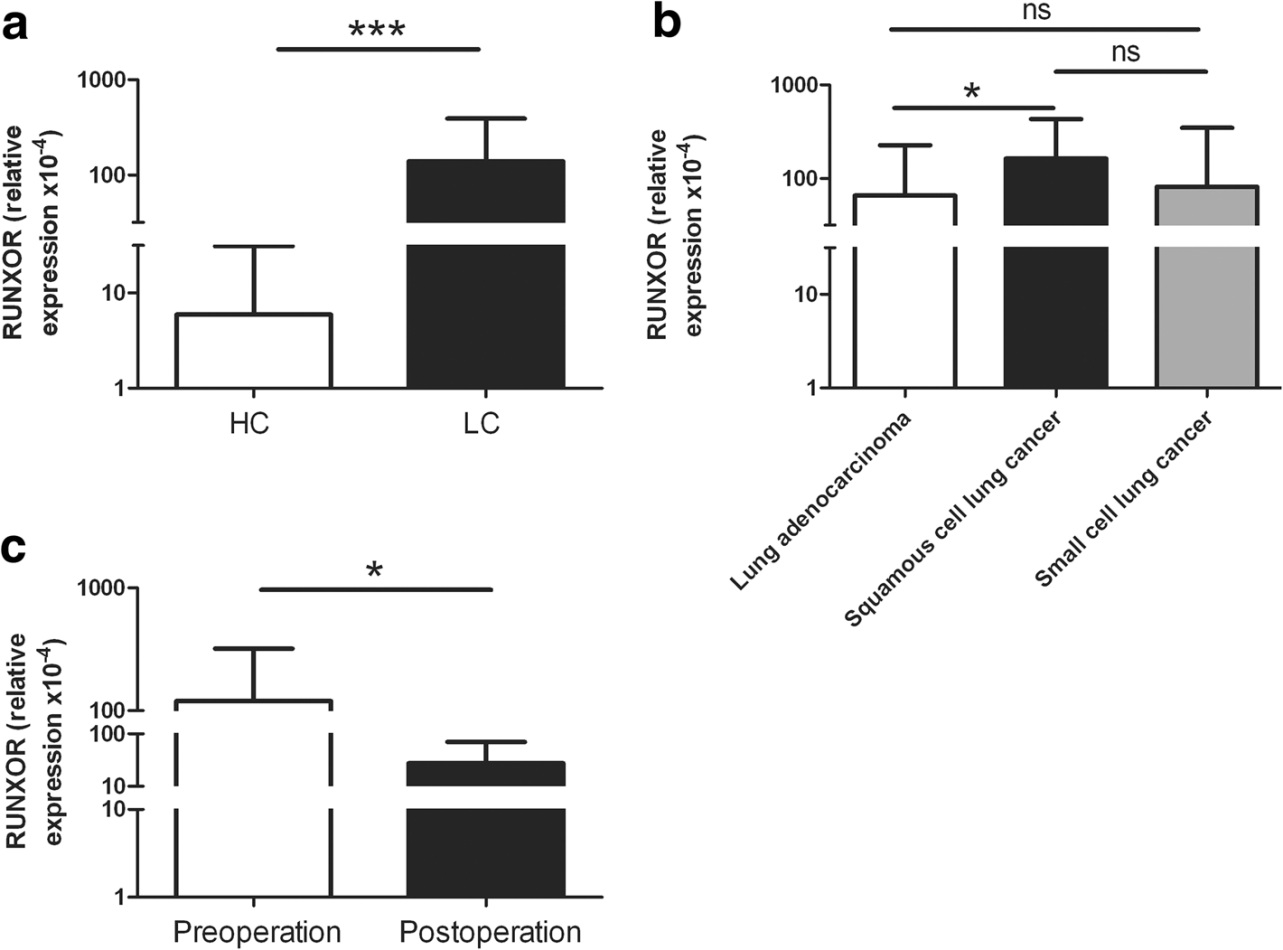
The level of lncRNA RUNXOR upregulated in lung cancer. **a** Relative expression of the lncRNA RUNXOR in the peripheral blood of lung cancer patients and healthy donors. **b** The relative expression of lncRNA RUNXOR in peripheral blood from different types of lung cancers. **c** The relative expression of lncRNA RUNXOR in the peripheral blood of lung cancer patients pre- and post-operation. ***P < 0.001, *P < 0.05, ns: no significance

We used real-time fluorescence quantitative PCR (qRT-PCR) to detect the expression of lncRNA RUNXOR in the peripheral blood of lung cancer patients. We found that compared with healthy controls, the RUNXOR level increased in the peripheral blood of lung cancer patients (*P* < 0.001, Fig. 2a). In addition, by analyzing the RUNXOR level in different types of lung cancers, we found that RUNXOR was differently expressed between squamous cell lung cancer and lung adenocarcinoma, which indicated that RUNXOR might be used to distinguish lung cancer types (*P* < 0.05, Fig. 2b). Interestingly, we found that RUNXOR expression was significantly downregulated in the blood of lung cancer patients who have underwent surgery (P < 0.05, Fig. 2c). Additionally, the data in Table 1 showed that the RUNXOR level was remarkably correlated with smoking history (*P* = 0.039), TNM stage (*P* < 0.0001), histological tumor type (*P* = 0.016) and lymph node metastasis (*P* = 0.0028) in lung cancer. These results show that the lncRNA RUNXOR level is closely related with lung cancer.

### The expression of RUNXOR is associated with the immunosuppression of MDSCs

**Fig. 3:**
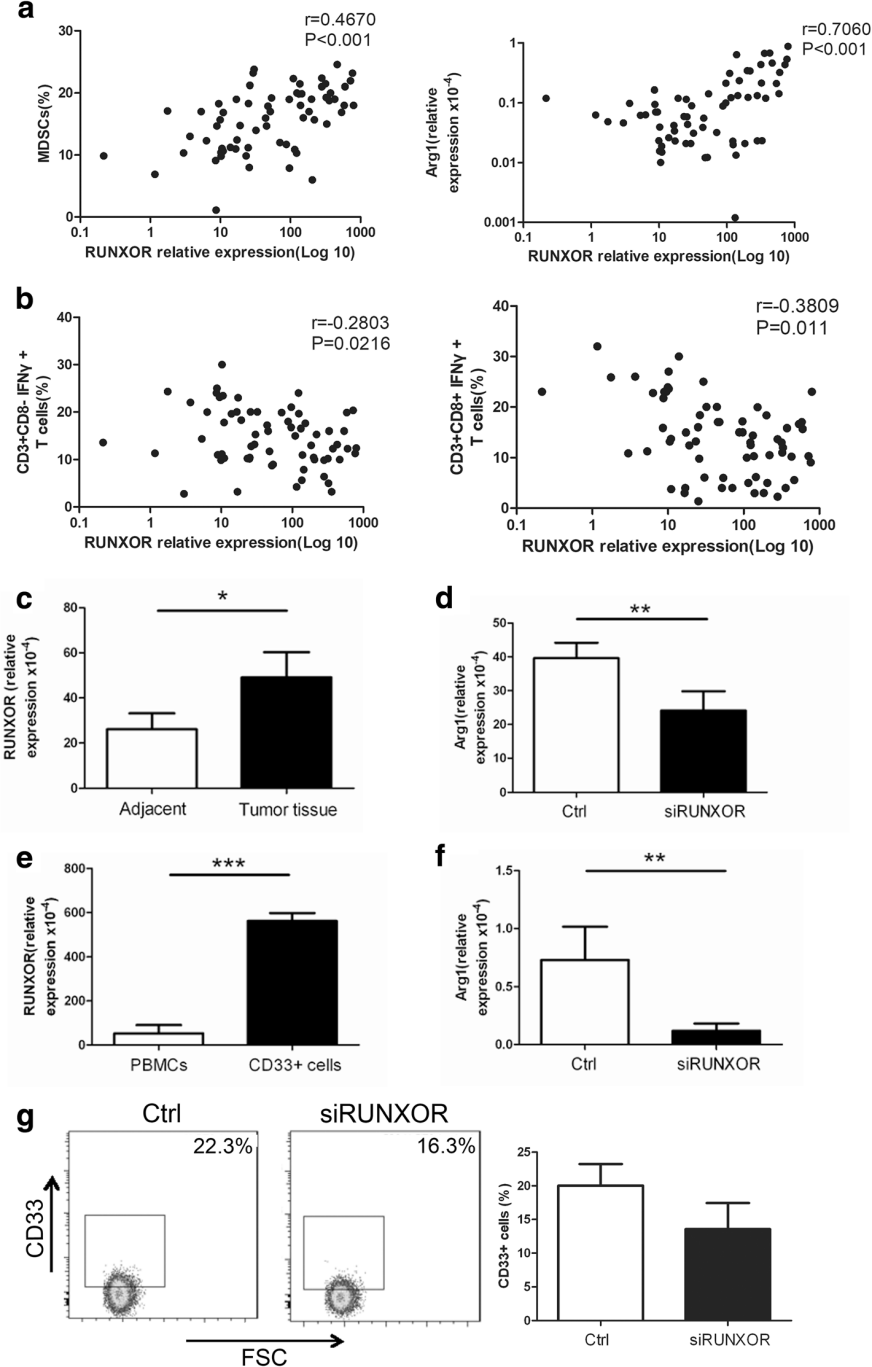
The expression of RUNXOR is associated with the immunosuppression of MDSCs. **a** The correlation between the expression of the lncRNA RUNXOR and the proportion of MDSCs and the expression of Arg1. **b** The correlation between the expression of lncRNA RUNXOR and the proportion of Th1/CTL cells. **c** The expression of lncRNA RUNXOR in MDSCs from tumor tissues of lung cancer patients. **d** The expression of Arg1 in MDSCs from tumor tissue of lung cancer patients after treatment with siRUNXOR. **e** The expression of lncRNA RUNXOR in CD33+ MDSCs induced from PBMCs of healthy donors. **f** The expression of Arg1 in induced MDSCs after treatment with siRUNXOR. **g** The effect of RUNXOR knockdown on the induction of CD33+ MDSCs. ***P < 0.001,**P < 0.01, *P < 0.05

Previous work has revealed that RUNXOR is significantly upregulated in AML which is characterized by the proliferation of immature myeloid cells [21]. As described above, RUNXOR is associated with lung cancer. Based on these findings, we hypothesized that RUNXOR might be involved in MDSC-induced immunosuppression in lung cancer. According to the correlation analysis, RUNXOR expression was positively correlated with the proportion of MDSCs and Arg1 level (Fig. 3a), which is the main suppressive molecule of MDSCs, in the peripheral blood of lung cancer patients. Meanwhile, RUNXOR expression was negatively correlated with the percentage of Th1 and CTL (Fig. 3b) cells in the peripheral blood of lung cancer patients. To further determine whether RUNXOR was expressed by MDSCs, we isolated CD11b + CD33 + HLA-DR-CD14- MDSCs from tumor and adjacent tissues of lung cancer patients and detected the expression of RUNXOR by using qRT-PCR. Compared with cells of the same phenotype from adjacent tissue, RUNXOR expression was increased in MDSCs from tumor tissue (*P* < 0.05, Fig. 3c). We also used a specific siRNA to inhibit the expression of RUNXOR and then detected the Arg1 level in MDSCs from tumor tissue. The production of Arg1 by MDSCs from tumor tissue was significantly downregulated (*P* < 0.01, Fig. 3d). We also used PBMCs from normal donors to induce MDSCs with GM-CSF + IL-1β and detected the expression of RUNXOR in the induced CD33+ MDSCs. We found that the RUNXOR level was increased in the induced MDSCs compared with PBMCs without stimulation (*P* < 0.001, Fig. 3e). Arg1 expression was clearly decreased in induced CD33+ MDSCs after RUNXOR knockdown (P < 0.01, Fig. 3f). To further verify whether RUNXOR could regulate the development of MDSCs from progenitor cells in the bone marrow, we detected the proportion of induced CD33+ cells in PBMCs post transfection with siRUNXOR, and found that the percentage of MDSCs decreased after knockdown of RUNXOR (*P* = 0.065, Fig. 3g). These data show that the expression of RUNXOR is positively correlated with MDSC-induced immunosuppression in lung cancer.

### The expression of RUNX1 is negatively correlated with the immunosuppression of MDSCs

**Fig. 4:**
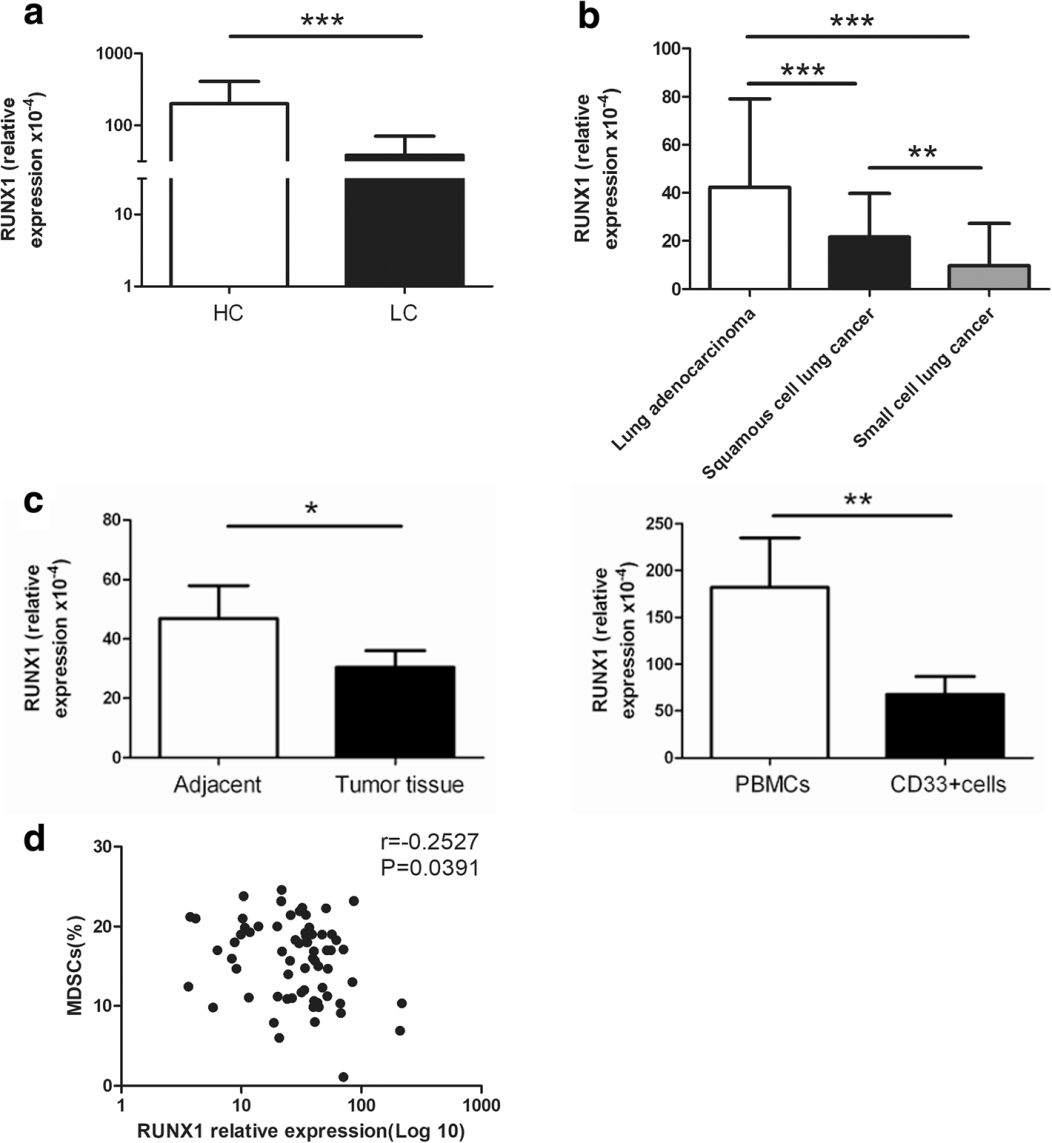
The expression of RUNX1 is negatively correlated with the immunosuppression of MDSCs. **a** The expression of RUNX1 in the peripheral blood of lung cancer patients and healthy donors. **b** The expression of RUNX1 in the peripheral blood of lung cancer patients with different histological categories. **c** The expression of RUNX1 in MDSCs isolated from the tumor tissues of lung cancer patients or induced from the PBMCs of healthy donors. **d** The correlation between the expression of the lncRNA RUNXOR and the proportion of MDSCs in the blood of lung cancer patients. (e) The correlation between the expression of the lncRNA RUNXOR and Arg1 in the blood of lung cancer patients. ***P < 0.001, **P < 0.01, *P < 0.05

RUNX1 is a critical molecule in the development of myeloid cells [26–28]. In our previous study, we confirmed that miR-9 regulates the immunosuppression and maturation of MDSCs by targeting RUNX1. In addition, RUNX1 is reported to be a target gene of RUNXOR, and RUNXOR is involved in the epigenetic regulation of RUNX1. RUNXOR interacts with the promoters and enhancers of RUNX1 gene via its 3′-terminal fragment. RUNXOR also participates in the formation of an intrachromosomal loop and then interacts with the H3-K27 methylase EZH2 and RUNX1 protein, which are known to regulate the gene function of RUNX1 [12, 21]. Here, we detected the expression of RUNX1 in the peripheral blood of lung cancer patients and found that compared with healthy controls, the RUNX1 level was decreased in lung cancer patients (*P* < 0.001, Fig. 4a). And RUNX1 level was the highest in the peripheral blood of patients with lung adenocarcinoma (Fig. 4b). In addition, RUNX1 expression in both the MDSCs from the tumor tissue of lung cancer patients and MDSCs induced with GM-CSF and IL-1β was decreased compared with cells of the same phenotype from adjacent tissue and PBMCs, respectively (*P* < 0.05, Fig. 4c). Meanwhile, we also showed that RUNX1 expression was negatively correlated with the proportion of MDSCs (Fig. 4d) in the peripheral blood of lung cancer patients. These results demonstrate that RUNX1 is negatively correlated with the immunosuppression of MDSCs.

### RUNXOR knockdown can increase the expression of RUNX1 in MDSCs

**Fig. 5:**
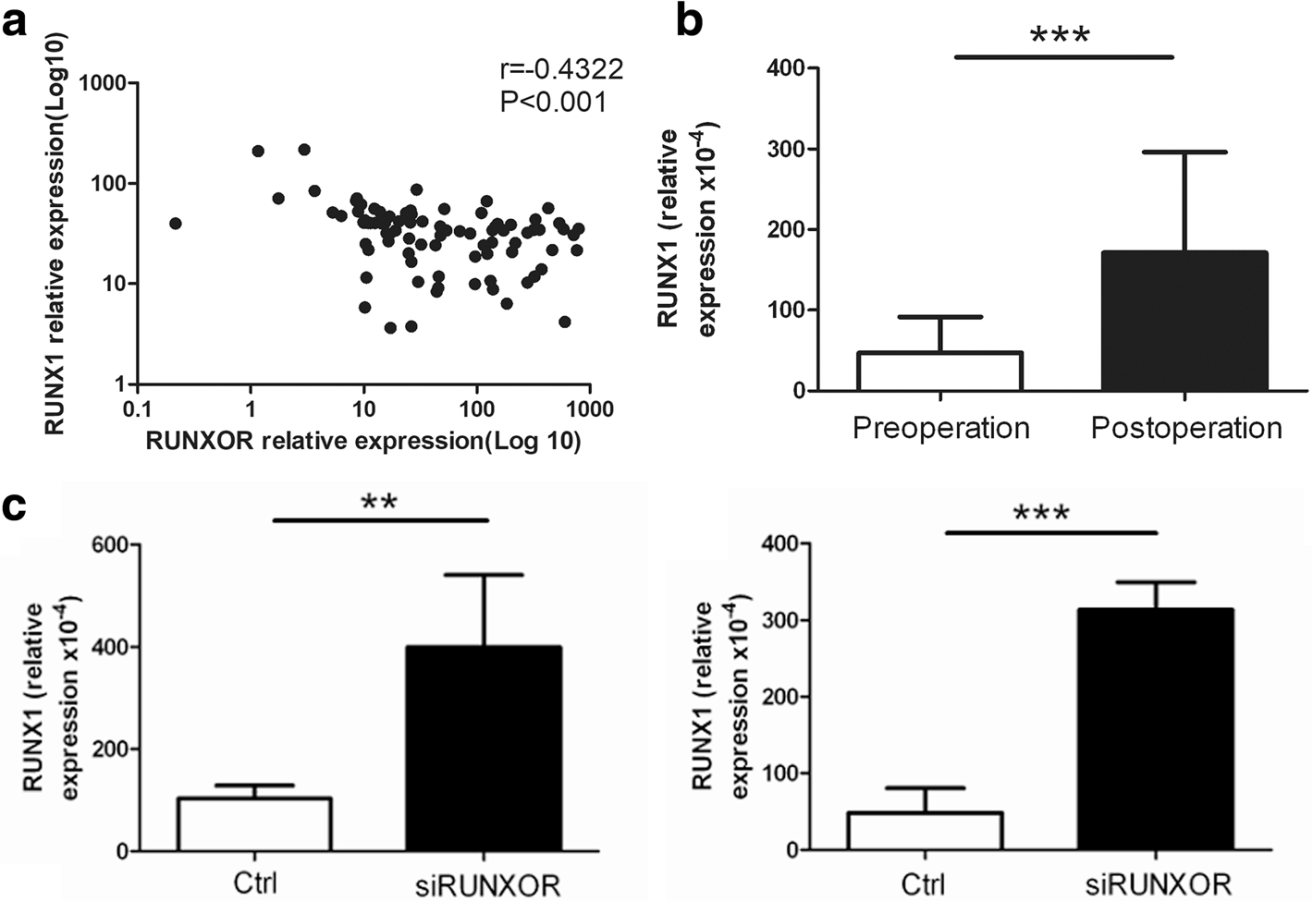
RUNXOR knockdown can increase the expression of RUNX1 in MDSCs. **a** The correlation between the expression of RUNXOR and RUNX1 in the peripheral blood cells of lung cancer patients. **b** The differential expression of RUNX1 in the blood samples of lung cancer patients pre- and post-operation. **c** The expression of RUNX1 in both MDSCs from the tumor tissue of lung cancer patients and MDSCs induced from PBMCs of healthy donors with RUNXOR knockdown. ***P < 0.001, **P < 0.01

To confirm whether RUNXOR regulates the expression of RUNX1 in MDSCs, we firstly analyzed the correlation between RUNXOR and RUNX1 and found that RUNXOR expression was negatively correlated with RUNX1 expression (Fig. 5a). In addition, the expression of RUNX1 increased after surgery in the peripheral blood of lung cancer patients, while the expression of RUNXOR decreased after surgery in the peripheral blood of lung cancer patients (*P* < 0.001, Fig. 5b). We next used a specific siRNA to interfere with RUNXOR expression and detected the RUNX1 expression in both MDSCs from the tumor tissue of lung cancer patients and MDSCs induced from healthy donor PBMCs with GM-CSF + IL-1β. After knockdown of RUNXOR, the expression of RUNX1 was upregulated in isolated and induced MDSCs (Fig. 5c). Thus, RUNXOR knockdown can increase the expression of RUNX1 in MDSCs.

## Discussion

LncRNAs play critical roles in various biological processes and diseases, including tumor progression [29–31]. Previous studies have demonstrated that RUNXOR, which overlaps with RUNX1, is a novel intragenic lncRNA that plays an important role in leukemogenesis [21]. In AML cells, RUNXOR directly binds to promoters and enhancers of the RUNX1 gene and participates in a long distance intrachromosomal interaction between two RUNX1 promoters. RUNXOR is also capable of facilitating the translocation of the RUNX1 gene. In addition, RUNXOR recruits epigenetic regulators, the EZH2 and RUNX1 proteins, to bind the promoter of the RUNX1 gene [21]. In this study, we demonstrated that the lncRNA RUNXOR is associated with the immunosuppression mediated by MDSCs, which consist of immature myeloid cells, in lung cancer patients.

The expression of lncRNA RUNXOR was detected in peripheral blood samples from lung cancer patients. Compared with healthy controls, RUNXOR expression in the peripheral blood of lung cancer patients was upregulated. In addition, the RUNXOR level was decreased in the blood of lung cancer patients after surgery. Since RUNXOR is found to be expressed in immature myeloid cells, we wondered whether it also exists in the MDSCs of lung cancer patients. We analyzed the correlation between RUNXOR expression and the proportions of MDSCs and Th1/CTL cells in the blood of lung cancer patients. The RUNXOR level was positively correlated with the MDSCs percentage and Arg1 level, while it was negatively correlated with the proportion of Th1/CTL cells, which indicated that RUNXOR expression may be involved in the immunosuppression of MDSCs in lung cancer patients. Thus, to confirm whether RUNXOR was expressed in MDSCs, we not only isolated CD11b + CD33 + HLA-DR-CD14- MDSCs from tumor tissue but also induced CD33+ MDSCs from PBMCs of healthy donors, and then detected the expression of RUNXOR in these MDSCs. We found that the expression of RUNXOR in MDSCs did increase. After RUNXOR knockdown, the expression of Arg1 in MDSCs was downregulated. To elucidate the mechanism by which RUNXOR regulates the immunosuppression of MDSCs, we detected the expression of a potential target gene of RUNXOR, RUNX1, in the blood samples of lung cancer patients and found that the RUNX1 level was downregulated in the peripheral blood of lung cancer patients compared with that of healthy controls. In addition, RUNX1 expression was restored in MDSCs when transfected with siRUNXOR, and RUNX1 was negatively correlated with the proportion of MDSCs from lung cancer patients. These data indicated that RUNXOR may affect the function of MDSCs via modulating RUNX1.

However, the exact regulatory mechanism by which RUNXOR regulates the suppressive function of MDSCs via targeting RUNX1 remains unclear. It has been shown that RUNXOR recruits EZH2 and RUNX1 to regulate the RUNX1 gene epigenetically in AML cells [21]. In addition, we previously demonstrated that miR-9 modulates the function and development of MDSCs by targeting RUNX1 [12]. Thus, we hypothesize that RUNXOR and miR-9 may cooperate to mediate the expression of RUNX1 at both the transcriptional and post-transcriptional level. In addition, we found that RUNXOR knockdown decreased the induction of MDSCs in vitro. These results indicate RUNXOR is associated with the development and immunosuppressive function of MDSCs in lung cancer.

## Conclusions

Taken together, our results indicate that RUNXOR is significantly associated with the immunosuppression induced by MDSCs in lung cancer patients and may be a target of anti-tumor immunity therapy.

## Abbreviations

AML: Acute myeloid leukemia
Arg1: Arginase 1
CTL: Cytotoxic T lymphocyte
EZH2: Enhancer of zeste homolog 2
GM-CSF: Granulocyte-macrophage colony-stimulating factor
IL-1β: Interleukin-1β
LncRNAs: Long non-coding RNAs
MDSCs: Myeloid-derived suppressor cells
M-MDSCs: Monocytic myeloid-derived suppressor cells
NcRNAs: Non-coding RNAs
PBMCs: Peripheral blood mononuclear cells
PMN-MDSCs: Polymorphonuclear myeloid-derived suppressor cells
RUNX1: Runt-related transcription factor 1
RUNXOR: RUNX1 overlapping RNA
Th1: T helper 1
